# One-step noninvasive prenatal testing (NIPT) for autosomal recessive homozygous point mutations using digital PCR

**DOI:** 10.1038/s41598-018-21236-w

**Published:** 2018-02-13

**Authors:** Mun Young Chang, Soyeon Ahn, Min Young Kim, Jin Hee Han, Hye-Rim Park, Han Kyu Seo, Jinsun Yoon, Seungmin Lee, Doo-Yi Oh, Changsoo Kang, Byung Yoon Choi

**Affiliations:** 1 Department of Otorhinolaryngology-Head and Neck Surgery, Chung-Ang University College of Medicine, 102 Heukseok-ro, Dongjak-gu, Seoul 06973 Republic of Korea; 2 Medical Research Collaborating Center, Seoul National University Bundang Hospital, 82 Gumi-ro 173 beon-gil, Bundang-gu, Seongnam 13620 Republic of Korea; 3 Department of Otorhinolaryngology, Seoul National University Bundang Hospital, 82 Gumi-ro 173 beon-gil, Bundang-gu, Seongnam 13620 Republic of Korea; 4 LAS Inc., 16 Arayuk-ro, Gimpo, 10136 Republic of Korea; 5 Bio-Medical Science Co., Ltd., BMS Bldg., 22 Yeoksam-ro 7-gil, Gangnam-gu, Seoul 06244 Republic of Korea; 6 Department of Biology and Research Institute of Basic Sciences, College of Natural Sciences, Sungshin Women’s University, Dongseon-dong 3-ga, Seongbuk-gu, Seoul 01133 Republic of Korea; 7 Wide River Institute of Immunology, Seoul National University College of Medicine, 101 Dabyeonbat-gil, Hwachon-myeon, Hongcheon 25159 Republic of Korea

## Abstract

Previously, we introduced a noninvasive prenatal testing (NIPT) protocol for diagnosing compound heterozygous autosomal recessive point mutations via maternal plasma DNA and simulated control genomic DNA sampling based on fetal DNA fraction. In our present study, we have improved our NIPT protocol to make it possible to diagnose homozygous autosomal recessive point mutations without the need to acquire fetal DNA fraction. Moreover, chi-squared test and empirical statistical range based on the proportion of mutant allele reads among the total reads served as the gatekeeping method. If this method yielded inconclusive results, then the Bayesian method was performed; final conclusion was drawn from the results of both methods. This protocol was applied to three families co-segregating congenital sensorineural hearing loss with monogenic homozygous mutations in prevalent deafness genes. This protocol successfully predicted the fetal genotypes from all families without the information about fetal DNA fraction using one-step dPCR reactions at least for these three families. Furthermore, we suspect that confirmatory diagnosis under this protocol is possible, not only by using picodroplet dPCR, but also by using the more readily available chip-based dPCR, making our NIPT protocol more useful in the diagnosis of autosomal recessive point mutations in the future.

## Introduction

Conventional methods for prenatal diagnosis have been amniocentesis and chorionic villus sampling, which carry a 1% risk of miscarriage^1–3^. Recently, noninvasive prenatal testing (NIPT) has been gaining popularity, as it only requires maternal peripheral blood^4,5^. NIPT is recommended as a primary trisomy screening test to all pregnant women^6,7^. To date, several methods of NIPT have been developed, according to various needs of patients^5,8–12^. With the development of various methods of NIPT, its overall convenience and accuracy have also been improving. In the previous study, we developed a novel protocol of NIPT applicable to autosomal recessive (AR) monogenic disease in predicting the genotype of a fetus (second baby) based on the first baby’s known genotype, using a higher-resolution picodroplet digital PCR (dPCR)^10^.

Although our previous protocol showed successful prediction, it required calculating the fraction of fetal DNA (the second baby’s fetal DNA) among the maternal plasma DNA (mpDNA) to produce simulated control samples and perform statistical analyses. In compound heterozygous genotypes, a fraction of fetal DNA can, if not always, be calculated by measuring the fraction of paternal mutation in mpDNA. However, a paternal allele-specific single nucleotide polymorphism (SNP), which does not exist in the maternal allele, should additionally be searched in homozygotes. However, this process is a time and effort-intensive task, which may not always be feasible. This process may be particularly difficult in east Asian populations where there are a lot of prevalent founder alleles, and therefore, homozygous genotypes for autosomal recessive disorders.

In our present study recruiting families segregating such homozygous AR deafness variants, we developed a novel, convenient NIPT protocol, which does not require either searching for a paternal allele specific SNP nor reconstruction of haplotypes. This protocol utilized both chi-squared test and Bayesian method, allowing for prenatal diagnosis without the calculation of fetal DNA fraction. This protocol successfully predicted fetal genotypes.

## Methods

### Subjects and Ethical Considerations

The institutional review boards of both Seoul National University Hospital (IRBY-H-0905–041–281) and Seoul National University Bundang Hospital (IRB-B-1007-105-402 and IRB-B-1508-312-304) approved all procedures used in this study. All subjects provided written informed consent. All methods were performed in accordance with the relevant guidelines and regulations. Three families with the first baby already confirmed to have SNHL due to AR mutations of known deafness genes and an unborn baby (fetus) were included in this study (Supplementary Figure S1). Causative mutation of SNHL from SH197 and SB275 families has previously been documented as *GJB2* c.235delC homozygote through bioinformatic analysis as described^13,14^. The causative mutation of SNHL from SH162 family has previously been documented as *SLC26A4* c.IVS7-2A > G. NIPT was performed for genotyping of the causative deafness gene from the unborn baby of each family.

### Plasma DNA extraction protocol

Blood samples were collected from all pregnant mothers. At the time of this procedure, the maternal gestational ages of SH197, SB275, and SH162 families were 16, 27 and 16 weeks, respectively. The maternal body weights were 48, 55, and 51 kg, respectively. Plasma DNA was extracted as described^10^; 1.5 ml plasma was added to a microcentrifuge tube and centrifuged for 10 min at 2000 g using MACHEREY-NAGEL, NucleoSpin Plasma XS (Germany) kit. Circulating DNA was extracted following the manufacturer’s guidelines.

### gDNA preparation

gDNA was prepared as described^15^. The control samples were made with gDNA previously obtained from the mother, and first baby in each family. To make the size of gDNA close to the size of plasma DNA, gDNA was fragmented using Covaris S220 (Covaris, MA, USA). The fragment size was confirmed as 150 base pair length by Bioanalyzer High Sensitivity DNA Chips (Agilent Technologies, CA, USA). DNA concentration was measured using a fluorescence assay of Picogreen (Invitrogen, Grand Island, NY, USA).

### Picodroplet digital PCR (dPCR) methods

Picodroplet dPCR was performed using RainDrop Digital PCR System (RainDance Technologies Inc., Billerica, MA, USA), as previously described^10^. PCR reaction mixes consisted of primers and probes (Supplementary Table S1 and Supplementary Figure S2) along with 12.5 µl TaqMan Genotyping Master Mix (Life Technologies), 1.25 µl Drop Stabilizer (RainDance Technologies), DNase/RNase-free sterile water, and template DNA (either the minimum 2 ng of plasma DNA or 30 ng of the fragmented gDNA), making up a total reaction volume of 25 µl. Each 25 µl PCR mix was emulsified into 5 pl droplet volumes using RainDrop Source instrument (RainDance Technologies). A single molecule of DNA was partitioned into approximately 5 million droplets. Then, PCR mixes were placed in a C1000 with deep-well (Bio-Rad) and amplified, according to the protocol (Supplementary Table S2). The fluorescent intensity of each droplet for two fluorophores (FAM and VIC) was identified using RainDrop Sense instrument (RainDance Technologies). The data from cluster plots were analyzed using RainDrop Analyst data analysis software, as previously described^10^.

### Chip-based dPCR methods

QX200 Droplet digital PCR System (Bio-Rad Laboratories, Inc., Hercules, CA USA) was used to assess nanodroplet dPCR. In a pre–polymerase chain reaction environment, PCR reaction mixes were combined with primers and probes (Supplementary Table S3) along with 10 μl TaqMan Genotyping Master Mix (Life Technologies), DNase/RNase-free sterile water, and template DNA (either the minimum 700 pg of plasma DNA or 30 ng of the fragmented gDNA), which made up a total reaction volume of 20 μl. A probe was validated (Supplementary Figure S2). Droplets were then generated using DG8 droplet generator cartridges by mixing the aqueous phase with 70 mL of droplet generation oil (Bio-Rad Laboratories). Each 20 μl PCR mix was emulsified into about 1 nl droplet volumes, partitioning a few molecules of DNA into approximately 20 thousand droplets. Droplets were transferred to a 96-well PCR plate and then sealed using the PX1 PCR plate sealer (Bio-Rad Laboratories) for 5 seconds at 180 degree before thermal cycling. The PCR plate was placed in a C1000 Touch thermal cycler with deep-well (Bio-Rad Laboratories) to be amplified, following the protocol outlined in Supplementary Table S4. After thermal cycling, the PCR plate that included droplets was loaded onto QX200 Droplet Reader instrument (Bio-Rad laboratories), identifying the fluorescent intensity of each droplet for two fluorophores (FAM and VIC) simultaneously using Multi-pixel photon counter. This detector reads the droplets to determine the ones that contain a target gene (+) and the ones that do not (−), and plots the fluorescence droplet by droplet. The fraction of positive droplets in a sample determines the concentration of target in copies/μl.

### Noninvasive prenatal testing (NIPT) protocol

**Figure 1:**
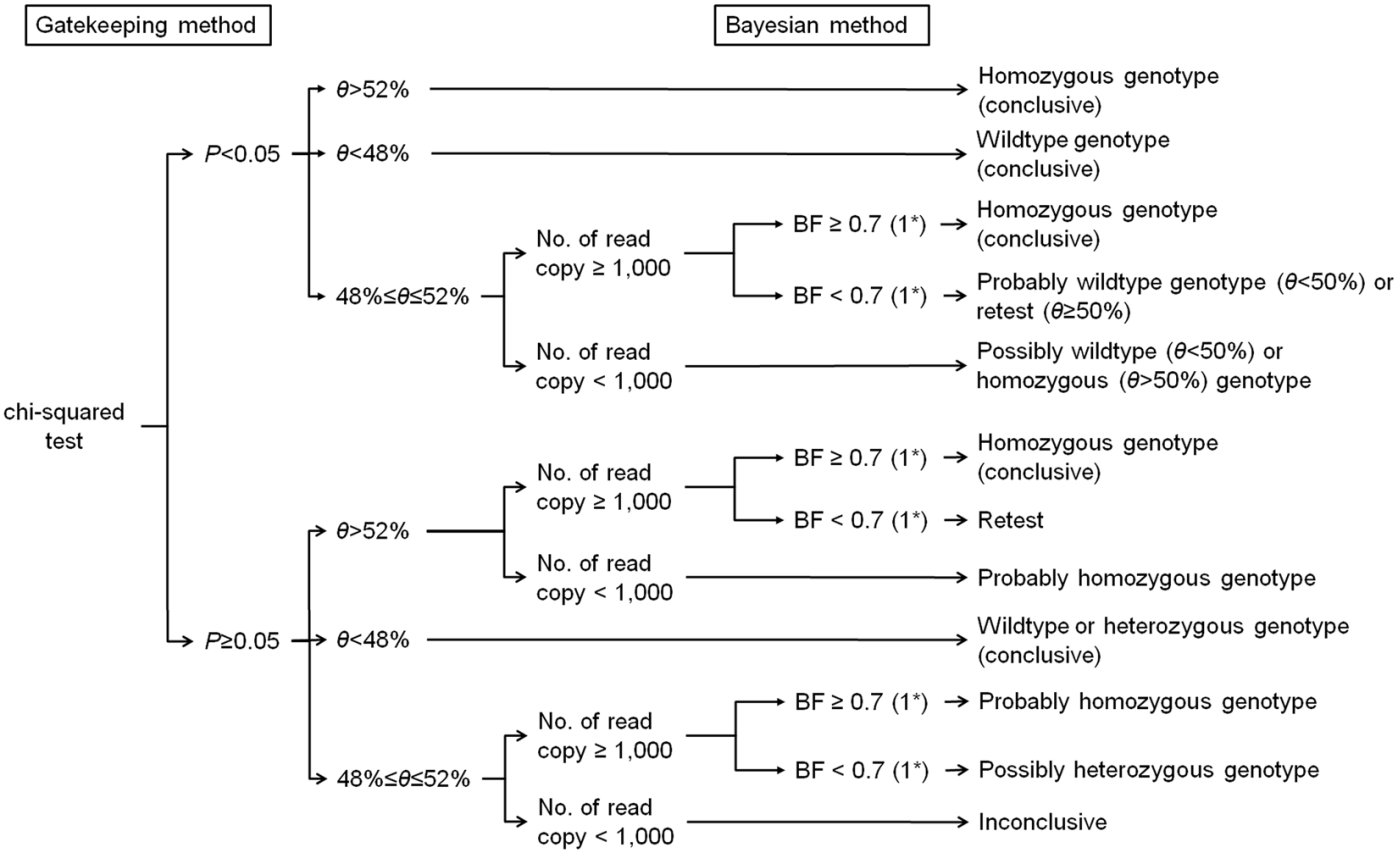
Protocol of noninvasive prenatal diagnosis. *The cutoff value of Bayes factor is 0.7 for picodroplet dPCR and 1 for chip-based dPCR. BF, Bayes factor.

We used both the gatekeeping method and Bayesian method to predict the genotype status of the fetus. The gatekeeping method was composed of chi-squared test and comparison of a proportion of the mutant allele reads among the total reads (*θ*) against the empirical range of the proportion from a heterozygous genotype, and it facilitated fast decision. The Bayesian method was based on prior information and data distribution, and robust decision was expected (Fig. 1).

In the gatekeeping method, chi-squared test of the plasma sample against the simulated heterozygous control was initially conducted. Maternal gDNA was used as the heterozygous control sample. When dPCR was performed on several samples from one subject, the observed mutant allele proportion was calculated by summing the mutant reads with the total droplet reads in each sample. The heterozygous control was expected to have a wild: mutant allele reads ratio of 50:50; rejecting the null hypothesis of chi-squared test indicated that the plasma sample is more likely to be either wild or mutant homozygote. To rule out any false negative results from chi-squared test due to errors and a small number of reads from dPCR, value of *θ* less than 48% or greater than 52% was regarded as a wild or mutant homozygous. This empirical range of the heterozygous genotype was obtained from a hypothetical range of the fetus DNA fraction (2.5–11%) and a statistical simulation assuming a normal experimental error rate of 1.0% (standard deviation 0.025%) and 1.5% (standard deviation 0.5%) for picodroplet and chip-based dPCR, respectively. The mean and standard deviation of error were obtained from simulated control with known a fetus DNA fraction. As the effect caused by the difference in the error rate between picodroplet and chip-based dPCR was not significant, the same range was applicable for both methods. Consequently, if a P-value from chi-squared test was less than 0.05 and the value of θ was less than 48% or more than 52%, the result was considered conclusive in the gatekeeping method. If not, the diagnosis was made considering a Bayesian method.

For the Bayesian method, a single droplet read was assumed to be generated from the Bernoulli trial. A mutant allele read was considered as a success. *θ* was necessarily affected by the amount of fetus DNA in the total plasma DNA (*p*). We introduced three genotype statuses of the fetus (*G*); a wildtype (*G* = 1) with *θ* = (1-*p*)/2, a single heterozygote (*G* = 2) with *θ* = 1/2, and a homozygous mutant (*G* = 3) with *θ* = (1 + *p*)/2. Prior information of three genotypes denoted as $\pi (G)$ was 0.25 (*G* = 1), 0.5 (*G* = 2), and 0.25 (*G* = 3), respectively. The priors imply the genotype is determined by the Mendelian inheritance. Prior information of *p*, $\pi (p)$, was assumed to follow the Beta distribution with α = 4 and ß = 100. It means that the prior mean of the amount of fetus DNA contained in the total plasma DNA was set as 4%. The observed mutant allele proportion of the *i*th sample was defined as mutant reads (*x*_*i*_) out of the total droplet reads (*n*_*i*_). When dPCR was performed on several samples from one subject, the observed mutant allele proportion was calculated by summing *x*_*i*_ and *n*_*i*_ in each sample. The posterior distribution of *G* can be calculated based on the experimental values as follows; $$\pi (p,\,G|x)\propto \,(\begin{array}{c}N\\ x\end{array})\,{(\theta (G,p))}^{x}{(1-\theta (G,p))}^{\{N-x\}}\times \,\pi (G)\times \pi (p)$$. We conducted the Bayesian hypothesis test using the Bayes factor, which is a ratio of two competing hypotheses; a higher value indicates the favorability of the alternative hypothesis over the null hypothesis. In our study, the null hypothesis was that the fetus was either a wildtype or a single heterozygote, whereas the alternative hypothesis was that the fetus was a homozygous mutant. If the Bayes factor of a homozygous mutant (*G* = 3) over a wildtype or a single heterozygote (*G* = 1 or 2) was greater than the cutoff value, the alternative hypothesis – homozygous mutant – was accepted.

To determine the cutoff value for the Bayes factor, we simulated both numerical and experimental studies. We made three types of biological control samples mimicking the wildtype, homozygous mutant, and heterozygote in each family. The homozygous mutant and wildtype control samples indicated the maternal gDNA with and without a homozygous mutation in a proportion of 5.6%, respectively. We used the first baby’s gDNA as the gDNA components with homozygous mutation. Maternal gDNA was used as the heterozygote control sample. Several datasets were computationally simulated based on the same assumption for the gatekeeping method; the error rate followed normal a distribution with a mean of 1.0% and a standard deviation of 0.025% for picodroplet dPCR and a mean of 1.5% and a standard deviation of 0.5% for chip-based dPCR; the various fetus DNA fraction ratios ranged from 2.5% to 11%. The cutoff value of Bayes factor was determined to be 0.7 for picodroplet dPCR and 1 for chip-based dPCR, since this cutoff provided 100% specificity and 90% sensitivity in our simulated data when the number of read copy was at least 1,000.

We developed a diagnostic protocol using both the gatekeeping method and Bayesian method. The former was first performed to obtain the quick diagnosis, if applicable. If the gatekeeping method yielded inconclusive results, the Bayesian method was conducted. Finally, results from both methods were considered (Fig. 1). Statistical analyses were performed using R 3.4.1.

### Confirmation of fetal genotype after birth

In the SH197 family, peripheral blood samples were obtained from the second baby after birth. Allele-specific PCR-based universal array (CapitalBio Corporation, Beijing, China) was applied to targeted the gene^11^. In SB275 and SH162 families, Sanger sequencing of gDNA from buccal mucosa of the second baby was performed to sequence the target residue after birth. The predicted fetal genotypes were checked against these results.

## Results

### Prediction of fetal genotypes by our NIPT protocol

In the SH197 and SB275 families, father and mother were single heterozygous carriers of *GJB2* c.235delC, and the first babies were homozygotes of c.235delC in *GJB2*. In the SH162 family, father and mother were single heterozygous carriers of *SLC26A4* IVS7-2A > G, and the first babies were homozygotes of IVS7-2A > G in *SLC26A4* (Supplementary Figure S1).

**Figure 2:**
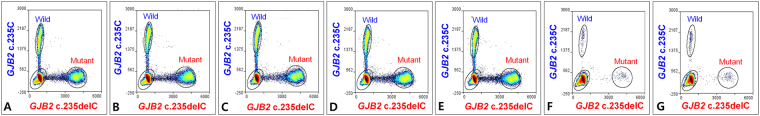
Two-dimensional histogram of the mutation (*GJB2* c.235delC) in wildtype (**A**), heterozygote (**B**,**C**) and homozygote (**D**,**E**) controls and maternal plasma DNA (**F**,**G**) of SH197 family.

In the SH197 family, a chi-squared test was conducted and *θ* of plasma sample was significantly lower than that of the heterozygous control (*P* = 0.001). *θ* of plasma sample (344/786 (43.77%)) was less than 48%. The fetus was diagnosed as a wildtype genotype (Tables 1 and 2 and Fig. 2

**Table 1:** The results of noninvasive prenatal testing using digital PCR.

Family	Probe	dPCR method	Sample	Total droplet	Mutant read	Total read	Corresponding histogram	Sum of mutant read	Sum of total read	Bayes factor
SH197	*GJB2* c.235delC	picodroplet dPCR	Wildtype control	4407215	11488	23677	Fig. 2(A)	11488	23677	0.000
			Heterozygote control	4345442	11437	23111	Fig. 2(B)	24188	48629	0.001
				4708179	12751	25518	Fig. 2(C)			
			Homozygote control	4617876	14615	28368	Fig. 2(D)	28844	56045	3488318818.000
				4630734	14229	27677	Fig. 2(E)			
			Maternal plasma DNA	3718365	149	342	Fig. 2(F)	344	786	-
				4441402	195	444	Fig. 2(G)			
SB275	*GJB2* c.235delC	picodroplet dPCR	Wildtype control	4152941	1355	2801	Supplementary Figure S3(A)	2729	5650	0.000
				4236265	1374	2849	Supplementary Figure S3(B)			
			Heterozygote control	4140496	1261	2537	Supplementary Figure S3(C)	1261	2537	0.083
			Homozygote control	3875232	1424	2771	Supplementary Figure S3(D)	2980	5781	4.802
				4174746	1556	3010	Supplementary Figure S3(E)			
			Maternal plasma DNA	4357569	521	981	Supplementary Figure S3(F)	1068	2007	14.885
				4410222	547	1026	Supplementary Figure S3(G)			
SH162	*SLC26A4* IVS7-2A > G	picodroplet dPCR, 1st trial	Wildtype control	4203431	2111	4487	Supplementary Figure S4(A)	3901	8294	0.000
				4044255	1790	3807	Supplementary Figure S4(B)			
			Heterozygote control	4063047	2330	4663	Supplementary Figure S4(C)	2330	4663	0.070
			Homozygote control	4295362	2821	5335	Supplementary Figure S4(D)	5409	10227	3304799.523
				4061939	2588	4892	Supplementary Figure S4(E)			
			Maternal plasma DNA	3460828	55	104	Supplementary Figure S4(F)	128	242	-
				4459482	73	138	Supplementary Figure S4(G)			
		picodroplet dPCR, 2nd trial	Wildtype control	4164784	1622	3444	Supplementary Figure S5(A)	1622	3444	0.000
			Heterozygote control	3877366	1613	3243	Supplementary Figure S5(B)	3700	7381	0.065
				4627099	2087	4138	Supplementary Figure S5(C)			
			Maternal plasma DNA	4040299	302	569	Supplementary Figure S5(D)	616	1160	2.889
				4130434	314	591	Supplementary Figure S5(E)			
		chip-based dPCR, 1st trial	Wildtype control	15688	1474	3014	Supplementary Figure S6(A)	1474	3014	0.020
			Heterozygote control	17114	1597	3214	Supplementary Figure S6(B)	2911	5871	0.024
				14247	1314	2657	Supplementary Figure S6(C)			
			Maternal plasma DNA	12516	135	257	Supplementary Figure S6(D)	135	257	-
		chip-based dPCR, 2nd trial	Wildtype control	14724	1600	3373	Supplementary Figure S7(A)	3302	6969	0.000
				14964	1702	3596	Supplementary Figure S7(B)			
			Heterozygote control	16493	1930	3965	Supplementary Figure S7(C)	1930	3965	0.007
			Homozygote control	16809	2016	3853	Supplementary Figure S7(D)	4034	7693	1944.177
				16040	2018	3840	Supplementary Figure S7(E)			
			Maternal plasma DNA	16253	65	115	Supplementary Figure S7(F)	139	246	-
				17601	74	131	Supplementary Figure S7(G)			

Maternal gDNA artificially containing the gDNA components without and with homozygous mutation in 5.6%, respectively, was used as the wildtype and homozygote control. The first baby’s gDNA was used as the gDNA components with homozygous mutation. Maternal gDNA was used as the heterozygote control sample. If the total read count was less than 1,000, the Bayes factor was not calculated. dPCR, digital PCR; gDNA, genomic DNA.

).

**Table 2:** Diagnostic results of noninvasive prenatal testing using digital PCR.

Family	dPCR method	Proportion of the mutant allele reads among the total reads in the plasma sample	Proportion of the mutant allele reads among the total reads in the heterozygous control sample	P-value of Chi-squared test	Proportion of the mutant allele reads among the total reads <48% or >52%	Bayes factor of plasma sample	Diagnosis
SH197	picodroplet digital PCR	344/786 (43.77%)	24,188/48,629 (49.74%)	0.001	Yes	—	Wildtype genotype
SB275	picodroplet digital PCR	1,068/2,007 (53.21%)	1,261/2,537 (49.70%)	0.018	Yes	14.885	Homozygous genotype
SH162	picodroplet dPCR, 1st trial	128/242 (52.89%)	2330/4663 (49.97%)	0.379	Yes	—	Probably homozygous genotype
	picodroplet dPCR, 2nd trial	616/1160 (53.10%)	3700/7381 (50.13%)	0.411	Yes	2.889	Homozygous genotype
	chip-based dPCR, 1st trial	135/257 (52.53%)	2911/5871 (49.58%)	0.389	Yes	—	Probably homozygous genotype
	chip-based dPCR, 2nd trial	139/246 (56.50%)	1930/3965 (48.68%)	0.020	Yes	—	Homozygous genotype

In the SB275 family, a chi-squared test was conducted and *θ* of plasma sample was significantly higher than that of the heterozygous control (*P* = 0.018). *θ* of plasma sample (1,068/2,007 (53.21%)) was more than 52%. The fetus was diagnosed as homozygous genotype (Tables 1 and 2 and Supplementary Figure S3). The prenatal diagnosis was obtained from the gatekeeping method, but the Bayesian method was conducted to verify the results from the gatekeeping method. The two plasma samples were tested, and the sum of mutant reads and total droplet reads were 1,068 and 2,007, respectively, resulting in a Bayes factor of 14.885. The fetus was diagnosed as homozygous genotype consistent with the result of the gatekeeping method (Tables 1 and 2 and Supplementary Figure S3).

In the SH162 family, we conducted several experiments using picodroplet and chip-based dPCR. According to our first result from picodroplet dPCR, *θ* of plasma sample showed no significant difference when compared with that of the heterozygous control (*P* = 0.379), although *θ* of plasma sample (128/242 (52.89%)) was more than 52%. As the number of read copy was less than 1,000, the Bayesian method was not conducted. The fetus was diagnosed as a homozygous genotype, without certainty (Tables 1 and 2 and Supplementary Figure S4). As this result was inconclusive, we conducted dPCR once more using picodroplet dPCR. According to our second result from picodroplet dPCR, *θ* of plasma sample showed no significant difference when compared with that of the heterozygous control (*P* = 0.411), although *θ* of plasma sample (616/1160 (53.10%)) was more than 52%. The Bayesian method was conducted, resulting in a Bayes factor of 2.889. The fetus was diagnosed as a homozygous genotype (Tables 1 and 2 and Supplementary Figure S5).

As for the chip-based dPCR, our first result showed that there was no significant difference of *θ* between the plasma sample and the heterozygous control (*P* = 0.389), although *θ* of plasma sample (135/257 (52.53%)) was more than 52%. As the number of read copy was less than 1,000, the Bayesian method was not conducted. The fetus was diagnosed as likely having a homozygous genotype (Tables 1 and 2 and Supplementary Figure S6). Given the inconclusive result, we conducted dPCR once more using chip-based dPCR. According to our second result from chip-based dPCR, *θ* of plasma sample was significantly higher than that of the heterozygous control (*P* = 0.018); *θ* of plasma sample (139/246 (56.50%)) was more than 52%. The fetus was diagnosed as homozygous genotype (Tables 1 and 2 and Supplementary Figure S7). The diagnosis using chip-based dPCR was consistent with using picodroplet dPCR.

### Genetic study for confirmation of fetal genotype

**Figure 3:**
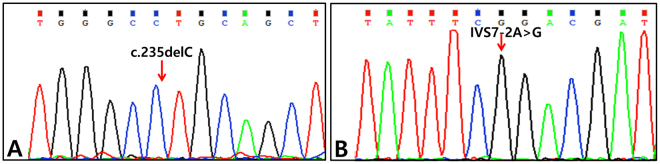
Genetic study for confirmation of fetal genotype. (**A**) Sanger sequencing traces of the second-born baby of SB275 family: *GJB2* c.235delC homozygote. (**B**) Sanger sequencing traces of the second-born baby of SH162 family: *SLC26A4* IVS7-2A > G.

In the SH197 family, allele-specific PCR-based universal array from the second baby confirmed a wildtype of *GJB2* c.235delC^16^. In the SB275 and SH162 families, Sanger sequencing from the second baby confirmed a homozygous mutant of *GJB2* c.235delC and *SLC26A4* IVS7-2A > G, respectively **(**Fig. 3**)**. The prenatal diagnosis for the second babies in all families was correct.

## Discussion

After discovering the cell-free fetal DNA (cffDNA) in the peripheral blood of pregnant women^17^, there has been tremendous development in the prenatal diagnosis using cffDNA^8,12,18^. The first NIPT using cffDNA was performed to determine fetal sex^17^ and RhD status^19,20^. The application of NIPT was extended from aneuploidies, such as trisomy 21, to monogenic diseases^8,10,18,21,22^. The expansion of NIPT applicability has been attributed to the improvement of technology and strategy of NIPT. The representative techniques of NIPT are massive parallel sequencing (MPS)^23^ and dPCR^8,12^.

NIPT using targeted MPS technology has also been performed by the reconstruction of fetal haplotypes, which required sequencing numerous single nucleotide polymorphisms (SNPs) around the residue of interest^24–28^. Recombination of fetal alleles has often made NIPT more complicated. Recently, it has been shown that the reconstruction of only the paired end allelic reads suffice for NIPT^4,5^. However, it is not possible to make the whole process of MPS technology for NIPT any simpler or shorter, regardless of the frequency of target mutation. The second NIPT technique using dPCR makes it possible to genotype the residue of interest directly. It may relatively be simpler and more straightforward than NIPT using MPS. Once we are well equipped with probes and primers for dPCR, with reaction conditions that have been well calibrated for certain founder mutations, then subsequent NIPT testing that targets a specific mutation may be easier and less time consuming to perform. Therefore, this technology is especially convenient for testing highly prevalent AR variants. Moreover, in our previous study, we showed that the accuracy of NIPT was further improved by utilizing picodroplet dPCR, which guarantees higher resolution^10^. However, there were procedural difficulties, especially in homozygous genotypes. In our previous protocol, prenatal diagnosis was determined by comparing the mutant fraction of the study sample with those of positive and negative controls^10^. The production of positive and negative controls was essential for the previous protocol, which can only be obtained via the calculation of the fraction of fetal DNA in mpDNA. In case that the maternal and the paternal mutation are different from each other, the fraction of fetal DNA in mpDNA can be calculated easily by measuring the fraction of the paternal mutant residue in mpDNA, if the fetus inherits the paternal mutation. However, in homozygous genotypes, both paternal and maternal mutations are identical; hence, the fraction of fetal DNA in mpDNA cannot be calculated simply by measuring the fraction of the paternal mutant residue in mpDNA. SNPs (more preferably, homozygous SNPs) that exist exclusively in the paternal gDNA, but not in the maternal gDNA, should be searched. This requires additional cost and time. To circumvent this issue, we developed a novel protocol for diagnosing the fetal genotype in homozygous genotypes without needing any information regarding the fraction of fetal DNA in mpDNA.

Theoretically, in determining genotypes of homozygous AR variants we can predict the genotype of a fetus based on the proportion of mutant allele reads among total reads in mpDNA: a proportion of mutant allele reads of less than 50% is considered a wildtype; a proportion of mutant allele reads that equals 50% is considered a single heterozygote; and a proportion of mutant allele reads of greater than 50% is considered a homozygote. However, since no test is perfectly precise, we must take in to account for any test errors. For example, we may consider two possibilities when a proportion of mutant allele reads is measured to be 50.5%. The first interpretation is that we can regard the proportion to have some experimental error and consider the genotype to be a single heterozygote. The second one is to consider the genotype to be a mutant homozygous, with a fairly small amount of fetus DNA in mpDNA. To distinguish these possibilities statistically, we employed a comprehensive simulation study for both the gatekeeping method and Bayesian method. We assumed the error rate to be within a normal distribution and a fetus DNA fraction to be within a range from 2.5% to 11%. The simulated data was used to determine an empirical range of mutant read proportion. The genotype of a fetus was expected to be wild or mutant homozygous when the proportion of mutant allele reads of mpDNA was smaller than 48% or greater than 52%, respectively, with a *P*-value from chi-squared test set to less than 0.05. We also generated simulated data for deciding the cutoff value for the Bayesian method. The Bayes factor cutoff value −0.7 for picodroplet dPCR and 1 for chip-based dPCR – was then tested for three types of control samples with known membership information and these proposed values worked successfully for prediction of genotypes of the control samples. Our numerical simulation showed that the proposed cutoff value was applicable to all cases with *GJB2* c.235delC and c.*SLC26A4* IVS7–2A > G mutation, under the condition that the total droplet read counts in dPCR is greater than or equal to 1,000 for statistical significance. Therefore, in the case of *GJB2* c.235delC and *SLC26A4* IVS7-2A > G, prenatal diagnosis can be made by examining only mpDNA in a single step, without further simulation. Expanding the scope further, if the cutoff value of Bayes factor is calculated for other prevalent homozygous mutations in this manner, a rapid diagnosis may likely be possible just from obtaining the maternal peripheral blood sample.

Another important implication of this study is that the improved NIPT protocol described in this paper successfully predicted the fetal genotypes using the chip-based dPCR platform. Although picodroplet dPCR has a higher resolution than chip-based dPCR, it is not widely available. If the NIPT protocol is only available using picodroplet dPCR, it may be difficult for most clinics to adopt this protocol, thus limiting the popularization. In this sense, our NIPT protocol coupled with chip-based dPCR and the gatekeeping method is expected to be more widely used and contribute to the popularization of NIPT.

As technology further develops, prenatal diagnosis will become more popular, with increased benefits. Our study shows that NIPT for all monogenic diseases could be easily performed by simply taking peripheral blood samples and performing quick statistical tests using the data generated from readily available chip-based dPCR, if the genotype of the first baby is available.

## Electronic supplementary material

Supplementary information
