# Supplementary material for: One-step noninvasive prenatal testing (NIPT) for autosomal recessive homozygous point mutations using digital PCR
Source: Sci Rep. 2018 Feb 13;8:2877. doi: 10.1038/s41598-018-21236-w (PMC5811538; doi:10.1038/s41598-018-21236-w)
Supplement: Supplementary file 1 — Supplementary information [file 41598_2018_21236_MOESM1_ESM.pdf]

Supplementary Information

One-step noninvasive prenatal testing (NIPT) for autosomal recessive homozygous point mutations using digital PCR

Mun Young Chang,[1*] Soyeon Ahn,[2*] Min Young Kim[3], Jin Hee Han[3], Hye-Rim Park[3], Han Kyu Seo[4], Jinsun Yoon[5], Seungmin Lee[3], Doo-Yi Oh[3], Changsoo Kang[6] & Byung Yoon Choi[3,7]

[1]Department of Otorhinolaryngology-Head and Neck Surgery, Chung-Ang University College of Medicine, 102 Heukseok-ro, Dongjak-gu, Seoul, 06973, Republic of Korea.

[2]Medical Research Collaborating Center, Seoul National University Bundang Hospital, 82 Gumi-ro 173 beon-gil, Bundang-gu, Seongnam, 13620, Republic of Korea.

[3]Department of Otorhinolaryngology, Seoul National University Bundang Hospital, 82 Gumi-ro 173 beon-gil, Bundang-gu, Seongnam, 13620, Republic of Korea.

[4]LAS Inc., 16 Arayuk-ro, Gimpo, 10136, Republic of Korea.

[5]Bio-Medical Science Co., Ltd., BMS Bldg., 22 Yeoksam-ro 7-gil, Gangnam-gu, Seoul 06244, Republic of Korea.

[6]Department of Biology and Research Institute of Basic Sciences, College of Natural Sciences, Sungshin Women's University, Dongseon-dong 3-ga, Seongbuk-gu, Seoul, 01133, Republic of Korea.

[7]Wide River Institute of Immunology, Seoul National University College of Medicine, 101 Dabyeonbat-gil, Hwachon-myeon, Hongcheon, 25159, Republic of Korea.

Correspondence to : Byung Yoon, Choi, MD, PhD
Department of Otorhinolaryngology, Seoul National University Bundang Hospital, 82 Gumi-ro 173 beon-gil, Bundang-gu, Seongnam 13620, Korea, Tel: +82-31-787-7406; Fax: +82-31-787-4057

Supplementary Table S1. The sequences and concentrations of primers and probes in picodroplet digital PCR.

Name	Sequence(5' to 3')	Conc. Used for RainDrop(μM)	Anneal Temp.
c.235delC-F	CCATCTCCCACATCCGGC	0.35	60
c.235delC-R	CACGTGCATGGCCACTAG	0.35	
c.235deC-WT Probe	VIC-AGCTGCAGGGCCCAT-NFQ	0.2	
c.235delC-MT probe	FAM-ATCAGCTGCAGGCCCAT-NFQ	0.2	

Conc, concentration; Temp, temperature.

Supplementary Table S2. The protocol of polymerase chain reaction in picodroplet digital PCR.

Step	Temp	Time	Cycles
Polymerase activation	95 ℃	10 min	1
Denaturation	95 ℃	15 sec	45
Annealing & Extension	*Depending on target	1 min	45
Incubation	98 ℃	10 min	1
Final Hold	12 ℃	Hold	

* A slow ramping speed (0.5◦C) was used during cooling from the denaturation step to the annealing step.

Temp, temperature.

Supplementary Table S3. The sequences and concentrations of primers and probes in chip-based dPCR.

Name	Sequence(5' to 3')	Conc. Used for QX200 (uM)	Anneal Temp.
SLC26A4_IVS7-2A>G -F	TTGACAAACAAGGAATTATTAAAACCAATGGA	0.35	
SLC26A4_IVS7-2A>G -R	TCCAGGTTGGCTCCATATGAAATG	0.35	62
SLC26A4_IVS7-2A>G -WT Probe	VIC-CAATTATCGTCTGAAATAA-NFQ	0.2	
SLC26A4_IVS7-2A>G -MT probe	FAM-ATCGTCCGAAATAA-NFQ	0.2	

Supplementary Table S4. The protocol of polymerase chain reaction in chip-based dPCR.

Step	Temp	Time	Cycles
Enzyme activation	95 ℃	10 min	1
Denaturation	94 ℃	30 sec	40
Annealing / Extension	62 ℃	1 min	
Enzyme deactivation	98 ℃	10 min	1
Cool down and storage	4 ℃	Hold	1

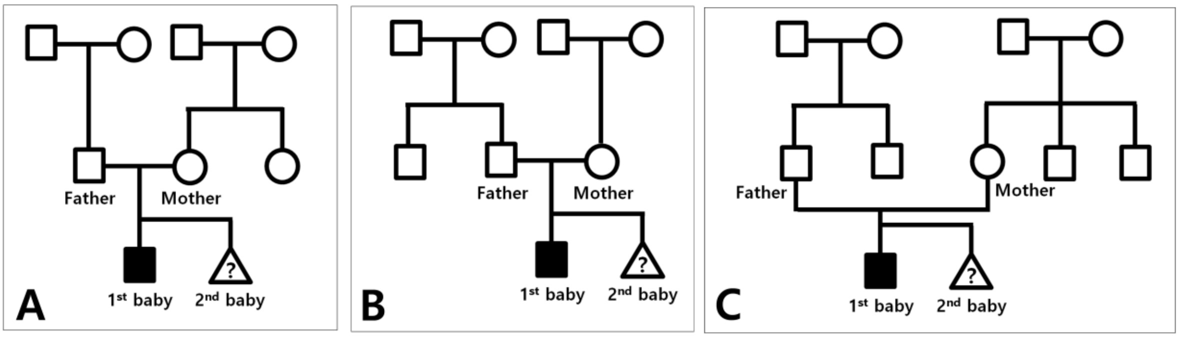

Supplementary Figure S1. Pedigree of three families. (A) Sanger sequencing traces of SH197 family. Father: *GJB2* c.235delC carrier, Mother: *GJB2* c.235delC carrier, the first baby: *GJB2* c.235delC homozygote. (B) Sanger sequencing traces of SB275 family. Father: *GJB2* c.235delC carrier, Mother: *GJB2* c.235delC carrier, the first baby: *GJB2* c.235delC homozygote. (C) Sanger sequencing traces of SH162 family. Father: *SLC26A4* IVS7-2A>G carrier, Mother: *SLC26A4* IVS7-2A>G carrier, the first baby: *SLC26A4* IVS7-2A>G homozygote.

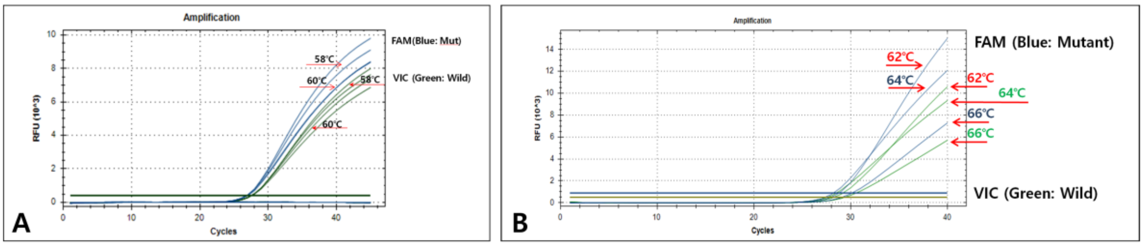

Supplementary Figure S2. Validation of probes. The target was detected after less than 40 cycles. (A) The probe for mutation in SH197 and SB275 families (*GJB2* c.235delC), (B) The probe for mutation in SH162 family (*SLC26A4* c.2168A>G).

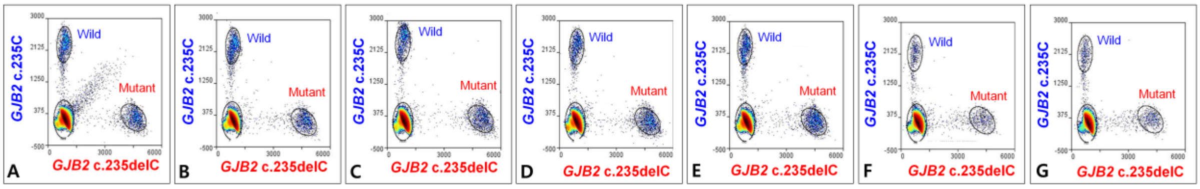

Supplementary Figure S3. Two-dimensional histogram of the mutation (*GJB2* c.235delC) in wildtype (A,B), heterozygote (C) and homozygote (D,E) controls and maternal plasma DNA (F,G) of SB275 family.

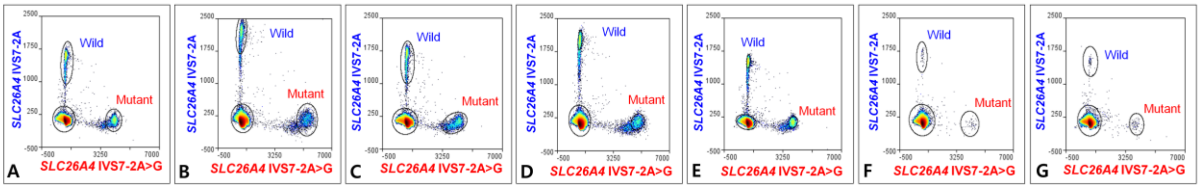

Supplementary Figure S4. Two-dimensional histogram of the mutation (*SLC26A4* IVS7-2A>G) in wildtype (A,B), heterozygote (C) and homozygote (D,E) controls and maternal plasma DNA (F,G) of SH162 family using picodroplet digital PCR (1st trial).

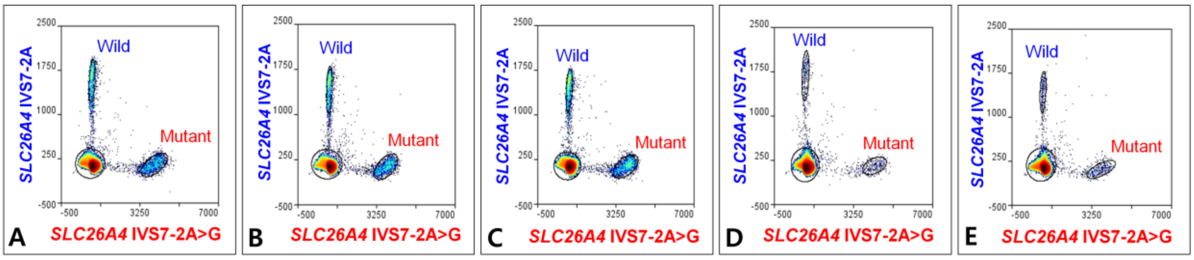

Supplementary Figure S5. Two-dimensional histogram of the mutation (*SLC26A4* IVS7-2A>G) in wildtype (A), heterozygote (B,C) controls and maternal plasma DNA (D,E) of SH162 family using picodroplet digital PCR (2nd trial).

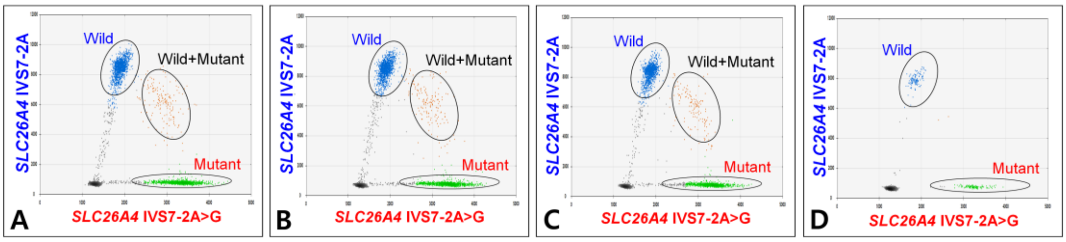

Supplementary Figure S6. Two-dimensional histogram of the mutation (*SLC26A4* IVS7-2A>G) in wildtype (A), heterozygote (B,C) controls and maternal plasma DNA (D) of SH162 family using chip-based dPCR (1st trial).

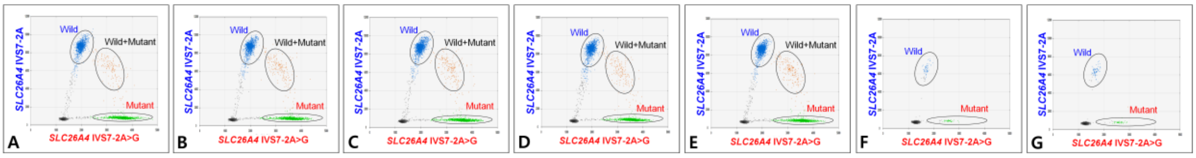

Supplementary Figure S7. Two-dimensional histogram of the mutation (*SLC26A4* IVS7-2A>G) in wildtype (A,B), heterozygote (C) and homozygote (D,E) controls and maternal plasma DNA (F,G) of SH162 family using chip-based dPCR (2nd trial).
